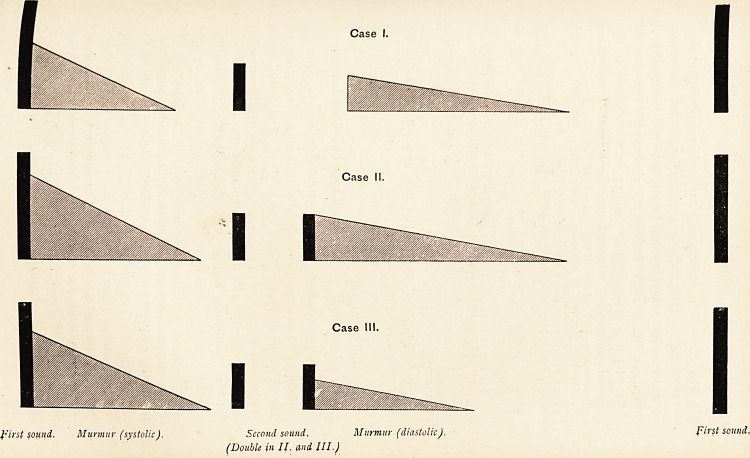# The Causation of Certain Mid-Diastolic Murmurs

**Published:** 1908-12

**Authors:** Carey Coombs

**Affiliations:** Assistant Physician to the Bristol General Hospital


### THE CAUSATION OF CERTAIN MID-DIASTOLIC MURMURS.

BY

CAREY COOMBS, M.D. Lond., M.R.C.P.,

*Assistant Physician to the Bristol General Hospital.*

THESE remarks will be best introduced by notes of three illustrative cases from my out-patient department.

**Case 1.**—A man, aged 26, with aortic regurgitation. Apex beat in sixth space outside the left mammary line. Cardiac dulness from one inch within the right mammary line to the left axilla. At the apex on some days one hears, in addition to a constant systolic bruit, a blowing murmur beginning in mid-diastole, not usually continuous with the next first sound. (See diagram.)

**Case 2.**—A woman, aged 26, with mitral regurgitation, and with signs such as raise a strong suspicion of pericardial adhesion. The area of impulse extends from the right sternal margin to the left axilla, and there is systolic recession of the left lower ribs behind. At the apex the sounds are as represented in the diagram; after a double second sound comes a rumbling mid-diastolic murmur.

**Case 3.**—A boy, aged 8, with chorea and the commonest type of rheumatic heart disease seen in childhood—enlargement of the left ventricle with mitral regurgitation. The diagram shows the sounds heard at the apex: the diastolic murmur shown was short, blowing, and strictly mid-diastolic.

Case I.

Case II.

Case III.

First sound.

Murmur (systolic).

Second sound.

(Double in II. and III.)

Murmur (diastolic).

First sound.

That diastolic murmurs are heard at the mitral area in cases of aortic regurgitation, adherent pericardium, and early rheumatic carditis is well known. Their origin, however, is a matter of dispute. This is partly because the similarity of the essential characteristics of these bruits has not been recognised. The following features of the murmur are common to all three cases :—

(1) The murmur is diastolic rather than presystolic, though sometimes it runs on to the next first sound.

(2) It is usually mid-diastolic, beginning either immediately after a double second sound or at a short interval after an undoubled second sound.

(3) It varies in length from day to day and even from beat to beat in the same patient.

(4) It is a smooth, blowing murmur, not rough and harsh like the presystolic murmur of mitral stenosis.

(5) Another point of difference from the murmur of stenosis is the absence of the crescendo character.

(6) It is best heard over a limited area above and internal to the point of maximum impulse.

(7) Sometimes it is accompanied by a fine diastolic thrill.

With so many points in common, it seems likely that, whether the case be one of aortic regurgitation, adherent pericardium, or earlier rheumatic heart disease, the origin of the apical diastolic murmur is always the same. The next step is to arrive at some plausible conception of this common origin.

Two factors are concerned in the production of an intracardiac bruit : a current of blood, and an orifice or channel modifying that current. The current of blood in these cases is a diastolic one. The apical position of the bruit enables us to set aside the possibility of its being due to incompetence of the semilunar valves, and we are therefore led to suppose that the vibrating stream must be that which flows from auricle to ventricle in the second half of diastole. The murmur is not crescendo, and it begins too soon in diastole to be connected with contraction of the auricle. It must, therefore, be connected with the suction action of ventricular diastole. Blood flows into the ventricle in diastole before the auricle has begun to contract, because the pressure within the ventricle has rapidly fallen as the ventricle expands. Where the ventricle is enlarged this fall in pressure will probably be greater, and the force of suction will therefore be more considerable. It is likely, then, that in the cases under discussion the suction exercised by an expanding ventricle is the force moving the current of blood whose vibrations are heard as a murmur.

We have yet to determine what is the influence by which vibrations are set up. We have already concluded that it is an auriculo-ventricular stream that is concerned: that it is the mitral stream is almost certain, because the murmur is heard in much the same position as that of mitral stenosis. Indeed, it might be thought that the murmur under consideration is produced by the same valvular changes as are found in mitral stenosis, perhaps in a less advanced stage. Such a theory is widely held, and it has two strong arguments in its favour. These are, first, that mitral valvular inflammation is generally present in cases of aortic valvular disease, adhesive pericarditis, and even in the earlier stages of rheumatic heart disease; and, second, that in cases of mitral stenosis a diastolic murmur is often heard at the apex even when the failure of auricular systole has blotted out the presystolic murmur. Both these facts seem to suggest that diastolic murmurs, in the conditions which we are considering, are always to be taken as direct proof that the mitral valve is becoming thick, fibrous and obstructive. *Post-mortem* evidence, however, disturbs this theory. The diastolic bruit heard at the apex in aortic regurgitation was first described by Austin Flint, because he was so surprised to find that it did not depend on narrowing of the mitral orifice; and it was in the same way that the corresponding murmur in adherent pericardium first attracted the attention of Graham Steell. As to the early rheumatic heart disease of childhood, it has twice been my fortune to make *post-mortem* examinations on cases where mitral stenosis had been diagnosed because of apical diastolic murmurs heard during life. Yet in both cases there was but the merest trace of mitral endocarditis, and in both the mitral opening was larger than normal.

Clearly, then, the murmur under discussion is not, at any rate, always proof of mitral valvular inflammation. Yet it must be at the mitral opening that the murmur is produced, and it remains for us to see whether there is any abnormal state of the mitral valve other than inflammation of the cusps, common to cases of aortic regurgitation, early rheumatic carditis, and adherent pericardium, by which an apical diastolic murmur could be caused.

One morbid change there is that is seen in the heart in all three diseases, and that is ventricular enlargement. The *cor bovinum* of aortic regurgitation, the enormous enlargement of the heart, which is the safest clinical guide to a diagnosis of adherent pericardium, the swollen heart of the rheumatism of childhood—in all these the greatest increase is in the ventricular capacity, especially in that of the left. Now if a portion of the ventricle be stretched C times in the course of a dilatation, the capacity of the ventricle will be increased $C_3$ times, but the calibre of the mitral channel will be increased $C_2$ times only.

More important still, the mitral ring will not even stretch $C_2$ times, because it is mainly built up of firm, strong, fibrous tissue, which is less readily influenced by mechanical or toxic influences than is muscle. When the left ventricle is enlarged, therefore, its cubic capacity is increased more than the size of the mitral ring. so that a relative narrowing of the mitral opening is in this way established. The diastolic murmurs heard at the apex in the conditions alluded to are, therefore, caused by vibrations in the blood as it is sucked into the expanding ventricle through a relatively small mitral opening.

This conception is of importance in practice because it explains the disappearance of such murmurs, at any rate in children, under the influence of rest, and also the long interval that may elapse between the first appearance of such a murmur in a rheumatic child and the development of true mitral stenosis—if, indeed, the latter develops at all.

To summarise :—

In aortic regurgitation, early rheumatic carditis, and adherent pericardium, diastolic murmurs are heard at the mitral area.

In their principal features these murmurs are so much alike as to suggest a common mode of production.

Autopsy shows that in all three diseases such murmurs may occur without valvular mitral stenosis being present.

Probably in such cases the bruit is due to vibrations in blood sucked by ventricular diastole into an enlarged ventricle, through a mitral orifice not so much enlarged.

In the discussion following this paper. Drs. Neild and Edgeworth suggested the use of this theory to explain murmurs apparently presystolic occurring under similar conditions in the absence of mitral valvular stenosis. With these suggestions I heartily agree. Possibly in such murmurs auricular systole plays its part, though not, I think, to the same extent as in the production of the presystolic murmur of true mitral stenosis.

## Figures and Tables

**Case I. Case II. Case III. f1:**